# Genomic predictive model for recurrence and metastasis development in head and neck squamous cell carcinoma patients

**DOI:** 10.1038/s41598-017-14377-x

**Published:** 2017-10-24

**Authors:** Ilda Patrícia Ribeiro, Francisco Caramelo, Luísa Esteves, Joana Menoita, Francisco Marques, Leonor Barroso, Jorge Miguéis, Joana Barbosa Melo, Isabel Marques Carreira

**Affiliations:** 1 Cytogenetics and Genomics Laboratory, Faculty of Medicine, University of Coimbra, 3000-354 Coimbra, Portugal; 2 CIMAGO - Center of Investigation on Environment Genetics and Oncobiology - Faculty of Medicine, University of Coimbra, 3000-354 Coimbra, Portugal; 3 Laboratory of Biostatistics and Medical Informatics, IBILI - Faculty of Medicine, University of Coimbra, 3000-354 Coimbra, Portugal; 4 Department of Dentistry, Faculty of Medicine, University of Coimbra, 3000-075 Coimbra, Portugal; 5 Stomatology Unit, Coimbra Hospital and University Centre, CHUC, EPE, 3000-075 Coimbra, Portugal; 6 Maxillofacial Surgery Department, Coimbra Hospital and University Centre, CHUC, EPE, 3000-075 Coimbra, Portugal; 7 Department of Otorhinolaryngology - Head and Neck Surgery, Coimbra Hospital and University Centre, CHUC, EPE, 3000-075 Coimbra, Portugal

## Abstract

The head and neck squamous cell carcinoma (HNSCC) population consists mainly of high-risk for recurrence and locally advanced stage patients. Increased knowledge of the HNSCC genomic profile can improve early diagnosis and treatment outcomes. The development of models to identify consistent genomic patterns that distinguish HNSCC patients that will recur and/or develop metastasis after treatment is of utmost importance to decrease mortality and improve survival rates. In this study, we used array comparative genomic hybridization data from HNSCC patients to implement a robust model to predict HNSCC recurrence/metastasis. This predictive model showed a good accuracy (>80%) and was validated in an independent population from TCGA data portal. This predictive genomic model comprises chromosomal regions from 5p, 6p, 8p, 9p, 11q, 12q, 15q and 17p, where several upstream and downstream members of signaling pathways that lead to an increase in cell proliferation and invasion are mapped. The introduction of genomic predictive models in clinical practice might contribute to a more individualized clinical management of the HNSCC patients, reducing recurrences and improving patients’ quality of life. The power of this genomic model to predict the recurrence and metastases development should be evaluated in other HNSCC populations.

## Introduction

Head and neck squamous cell carcinoma (HNSCC) is the sixth most common type of cancer worldwide^1^. The overall five-year survival rate remains at approximately 50% even with treatment advances^2^. HNSCC patient outcomes are strongly linked to tumor stage. Patients with early stage tumors (I and II) have 60–95% possibility of successful treatment; however, when diagnosed two thirds of patients already exhibit disease in advanced stage (III and IV)^3^. Tumor recurrence and metastasis lead to a poor prognosis and quality of life, being the recurrence rate in HNSCC patients of about 50% during the first 2 years after the diagnosis of the primary tumor^4^. Patients with failure after first-line therapy have a median overall survival of less than 1 year^5^. Some clinical-pathological parameters have been pointed out to prognosis, recurrence, and survival, namely tumor primary site, nodal involvement, tumor thickness, and the status of the surgical margins^6^. However, in actual HNSCC clinical practice, treatment modalities and prognosis are still based only in the TNM staging system classification, which leads to a homogeneous treatment for different HNSCC tumors. Genomic factors also play an important role in the aetiology of these tumors, being the malignant transformation of the cells characterized by a progressive and sequential acquisition of genomic abnormalities, which provide a selective growth advantage to cancer cells. Accurate and reliable methods to predict which HNSCC patients are most likely to recur or to develop distant metastases would significantly enhance the choice of personalized treatment modalities and consequently improve survival of patients. This stratification of patients has been difficult to obtain due to the numerous anatomic sites, the unpredictable clinical behavior and heterogeneous molecular features of these tumors^7^. In this study, we used whole genome copy number alterations (CNAs) to predict recurrence/metastasis development in HNSCC patients. Our predictive model presented an accuracy of more than 80% and it was validated in a TCGA cohort, representing a step further in the identification of clinically significant biomarkers with predictive value for HNSCC management.

## Material and Methods

### Study population

The study protocol was approved by the Committee on Ethics in Research of the Faculty of Medicine of the University of Coimbra. All patients provided their written consent to participate in the study after being informed about the research purposes. The study was performed in accordance with the relevant guidelines and regulations.

The study cohort includes tissue specimens from 104 HNSCC patients who underwent treatment with curative intent. The patients were recruited between October 2010 and August 2015 from the Maxillofacial Surgery and the Department of Otorhinolaryngology - Head and Neck Surgery, of the Coimbra Hospital and University Centre, CHUC, EPE, Portugal. Diagnosis and staging were performed in accordance with the American Joint Committee on Cancer TNM staging system. The participants in this study answered a survey regarding lifestyle and risk factors for upper aerodigestive tract malignancies, including alcohol and tobacco consumption. Patients were followed-up through hospital revisits during routine clinical appointments. The final date of follow-up was February 29, 2016. The follow-up periods ranged from 6 to 64 months. The median follow-up time of our cohort was 18 months. Details of our study cohort are listed in Table 1.

**Table 1:** Clinic-pathologic characteristics of study population - our cohort.

Patients (n = 104)			
	n (%)		n (%)
Gender		Age at diagnosis (Years)	
**Male**	88 (84,6)	**<60**	52 (50)
**Female**	16 (15,4)	**≥60**	52 (50)
Anatomic Subsite		Invasion peri(neural)	
**Tongue**	44 (42,3)	**Yes**	47(45,2)
**Floor of the mouth**	28 (26,9)	**No**	40(38,5)
**Retromolar Trigone**	8 (7,7)	NA	17(16,3)
**Jugal Mucosa**	6 (5,8)	Differentiation	
**Palate**	4 (3,8)	**Well**	76 (73,1)
**Alveolar ridge**	6 (5,8)	**Moderate**	22 (21,2)
**Tonsil**	2 (1,9)	**Poor**	1(1,0)
**Larynx**	2 (1,9)	**NA**	5 (4,8)
**Hypopharynx**	2 (1,9)	Margins	
**Epiglottis**	1 (1,0)	**R0**	59(56,7)
**Supraglottis**	1 (1,0)	**R1**	28(26,9)
Tobacco		**NA**	17(16,3)
**Yes**	76 (73,1)	HPV	
**No**	28 (26,9)	**Positive**	3(2,9)
Alcohol		**Negative**	101(97,1)
**Yes**	70 (67,3)	Treatment	
**No**	31 (29,8)	**surgery alone**	33 (31,7)
**NA**	3 (2,9)	**Surgery** + **RT**	42 (40,4)
TNM stage		**Surgery** + **RT** + **QT**	13 (12,5)
**I**	18 (17,3)	**Surgery** + **QT**	1 (1,0)
**II**	27 (26,0)	**RT** + **QT**	12 (11,5)
**III**	20 (19,2)	**RT alone**	3 (2,9)
**IV**	39 (37,5)	Vital status	
		**Relapses/Metastasis in follow-up**	40 (38,5)
		**Dead - OSCC**	33 (31,7)
		**Dead-non-OSCC**	6 (5,8)
		**Alive**	65 (62,5)

For control, gingival tissues from healthy donors subjected to wisdom teeth removal were used.

The tissue samples were snap-frozen in liquid nitrogen within 30 min after resection and stored at −80 °C until use.

### DNA extraction and array-CGH analysis

DNA from fresh frozen tissues of patients and controls were extracted using a High Pure PCR Template Preparation Kit (Roche GmbH, Mannheim, Germany), according to the manufacturer’s instructions. The DNAs were quantified by UV spectrophotometric analysis using a Nanodrop 1000 Spectrophotometer (Thermo Scientific, USA).

High-resolution whole genome analyses were performed using Agilent SurePrint G3 Human Genome microarray 180 K (Agilent Technologies, Santa Clara, CA, USA), according Pinto-Leite *et al*. 2014^8^. DNA of tumor samples was labelled with Cy5 by random primer labelling. DNA from controls was labelled with Cy3. Results were analysed using Agilent Genomic Workbench v6.5 software with the following settings: ADM1 as aberration algorithm, threshold of 6.0, moving average 2 Mb. The results are according to Human Genome build 19 and include imbalances with at least three consecutive probes with abnormal log_2_ ratios.

### Validation cohort from TCGA data portal

Copy number data, obtained by SNP array using Affymetrix Genome-Wide Human SNP Array 6.0 were downloaded from The Cancer Genome Atlas (TCGA) Data Portal along with the patients’ clinical data of 95 HNSCC, available at https://tcga-data.nci.nih.gov/tcga/, on the 23rd October, 2015. The available copy number data was Level 3 data.

Tissue samples were collected by TCGA with appropriate informed consent from newly diagnosed HNSCC patients at the time of their surgical resection. Human Genome Version 19 samples without germline CNVs were used.

Only copy number information for tumour samples that came from the anatomical locations of the tumours contained in our cohort was selected. In addition, only patients that had available recurrence/metastasis status information were considered. The median follow-up time of the TCGA cohort was 22 months. The clinical-pathologic features of the validation cohort are listed in Table 2.

**Table 2:** Clinic-pathologic characteristics of study population - TCGA cohort.

Patients (n = 95)			
	n (%)		n (%)
Gender		Age at diagnosis (Years)	
**Male**	67 (70.5)	**<60**	40 (42.1)
**Female**	28 (29.5)	**≥60**	55 (57.9)
Anatomic Subsite		Invasion peri(neural)	
**Tongue**	44 (46.3)	**Yes**	49 (51.6)
**Floor of the mouth**	13 (13.7)	**No**	34 (35.8)
**Buccal Mucosa**	6 (6.3)	NA	12 (12.6)
**Palate**	5 (5.3)	Margins	
**Alveolar ridge**	7 (7.4)	**R0**	74 (77.9)
**Oral Cavity**	20 (21.1)	**R1**	7 (7.4)
Tobacco		**NA**	14 (14.7)
**Yes**	70 (73.7)	Treatment	
**No**	24 (25.3)	**NA**	72 (75.8)
**NA**	1 (1.1)	**Surgery** + **RT + QT**	1 (1.1)
Alcohol		**Surgery** + **QT**	5 (5.3)
**Yes**	67 (70.5)	**RT** + **QT**	14 (14.7)
**No**	27 (28.4)	**RT alone**	3 (3.2)
NA	1 (1.1)	Vital status	
TNM stage		**Relapses/Metastasis in follow-up**	27 (28.4)
**I**	6 (6.3)	**Dead - OSCC**	17 (17.9)
**II**	18 (18.9)	**Dead-non-OSCC**	4 (4.2)
**III**	18 (18.9)	**Alive**	69 (72.6)
**IV**	53 (55.8)	**NA**	5 (5.3)

### Statistical analysis

#### Data Preparation

Chromosomes were binned by mean size of alterations in each chromosome, both in our cohort and the TCGA patients. Then, the alterations present in the patients were distributed by those bins, reducing significantly the number of regions to analyse as well as generating a more structured dataset that can be compared across cohorts.

#### Statistical Classification

Both our cohort and the cohort of patients from TCGA Data Portal were analysed. The latter was used as a means of external validation.

A three-class support vector machine (SVM) algorithm for statistical classification was applied to these data. The three studied classes were: patients with recurrence/metastasis, patients without recurrence/metastasis and patients of unidentifiable class.

The most important regions for the distinction between classes were selected by Gini’s coefficient given by a Variable Importance Plot in a bootstrapping scheme applied to a balanced set regarding the number of cases in each class. Since the number of observations in both data sets was limited, few genomic regions were used as well: the six most important variables, for a minimum number of 32 cases, were selected.

The final multiclass classifier is obtained by the combination of three binary classifiers that distinguishes between: i) having or not recurrence/metastasis; ii) not having recurrence/metastasis and being unidentifiable; iii) having recurrence/metastasis and being unidentifiable. The three binary classifiers are applied to data and their responses are combined using a voting strategy, thus obtaining the final classification.

Model performance is reported across 5000 iterations, executed twice. The algorithm’s performance was evaluated by the accuracy considering balanced sets. All analyses were performed using R (version 3.4.0) and Matlab (R2016b).

## Results

### CNAs detection in HNSCC cohort

**Figure 1:**
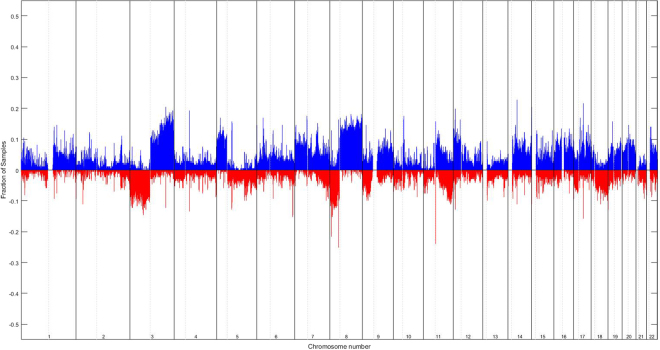
Profile of chromosomal imbalances detected in HNSCC patients using array-CGH technique. Blue represents copy number gains and red copy number losses. The fraction of samples means the fraction of patients that exhibited the imbalance. In X axis, chromosome number, from p arm to q arm (right to left), nucleotide position is represented.

The genomic characterization of HNSCC through whole genome array-CGH revealed several copy number gains and losses in all chromosomes (Fig. 1), being the chromosomes 3, 5, 7, 8, 9, 11, 12, 14, 15, 16, 17, 18, 19, 20 and 22 the most frequently altered. The most frequent copy number gains were observed at chromosomes 3q, 5p, 7p, 7q, 8q, 11q, 12p, 14, 15, 16, 17, 19, 20, 22 and the most frequent copy number losses were observed at chromosomes 3p, 8p, 9p, 11qter and 18 (Fig. 1).

### Development of a genomic predictive model for HNSCC recurrence and metastasis

The identified chromosomal alteration profile of our HNSCC patients together with their follow up clinical data (6–64 months of clinical follow up) were used to build a predictive model for HNSCC recurrence/metastasis development. This model includes three phases: i) identification of patients with vs. without recurrence/metastasis developed after the diagnosis and treatment of primary tumor, during follow up ii) distinction between patients without recurrence and unidentifiable, iii) distinction between patients with recurrence and unidentifiable.

This three-phase model presented an accuracy of 83.6% CI95% [66.7; 94.4]%, correct prediction of patients without recurrence/metastasis of 92.3% CI95% [66.7; 100]%, correct prediction of patients with recurrence/metastasis of 87.0% CI95% [50.0; 100]% and correct prediction of patients unidentified of 71.4% CI95% [33.0; 100]%.

This model was developed in three phases since we observed that in the first phase of the classification some patients were frequently and systematically misclassified. Interestingly, these misclassified patients presented an overall genomic profile similar to the patients that developed recurrence/metastasis (data not shown). After this observation, we decided to develop two new phases in this predictive model, where we considered the misclassified patients in the first phase of the predictive model as a new category labeled as unidentifiable patients.

**Figure 2:**
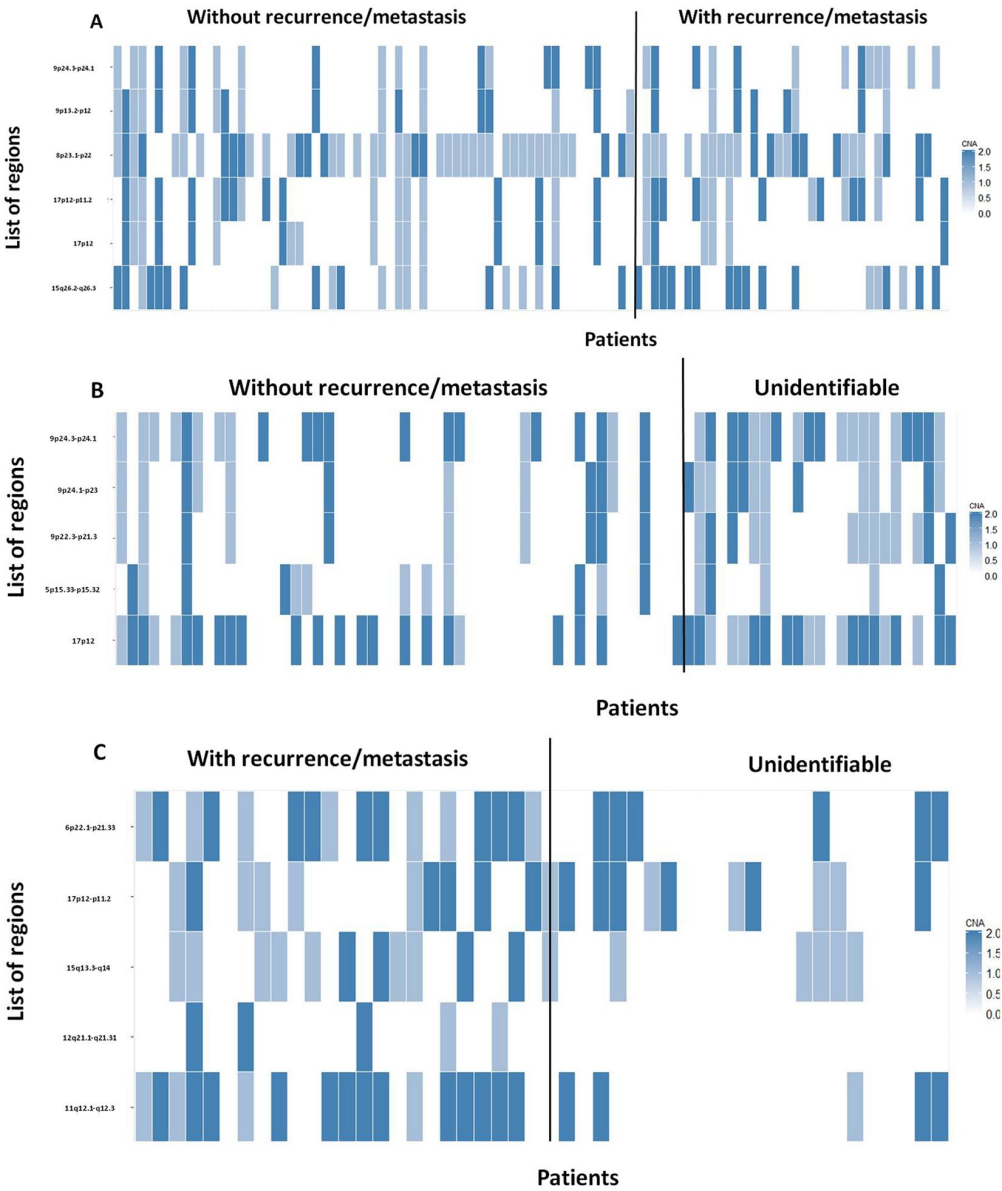
Heatmap with copy number alteration profile in the chromosomal regions used by the different phases of the predictive genomic model, (**A**) in patients with vs. without recurrence/metastasis - first phase of the predictive model; (**B**) in patients without recurrence and those unidentifiable - second phase of the predictive model; (**C**) in patients with recurrence and those unidentifiable - third phase of the predictive model.

This predictive model, in the first phase: identification of patients with vs. without recurrence/metastasis used six chromosomal regions: 8p23.1-p22, 9p13.2-p12, 9p24.3-p24.1, 15q26.2-q26.3, 17p12 and 17p12-p11.2 (Fig. 2A).

The second (patients without recurrence vs. unidentifiable) and third (patients with recurrence vs. unidentifiable) phases of this predictive model considered the following specific chromosomal regions, 8p23.1-p22, 9p13.2-p12, 9p24.3-p24.1, 15q26.2-q26.3, 17p12, 17p12-p11.2 and 6p22.1-p21.33, 11q12.2-q12.3, 12q21.2-q21.31, 15q13.3-q14 and 17p12-p11.2, respectively (Fig. 2B,C).

The bands 9p24.3-p24.1 and 17p12 are important for the discrimination of patients that developed or not recurrence/metastasis and for patients without recurrence/metastasis and the unidentifiable ones.

The band 17p12-p11.2 is important for the discrimination of patients that developed or not recurrence/metastasis as well as of patients that develop recurrence/metastases from those that are unidentifiable.

**Figure 3:**
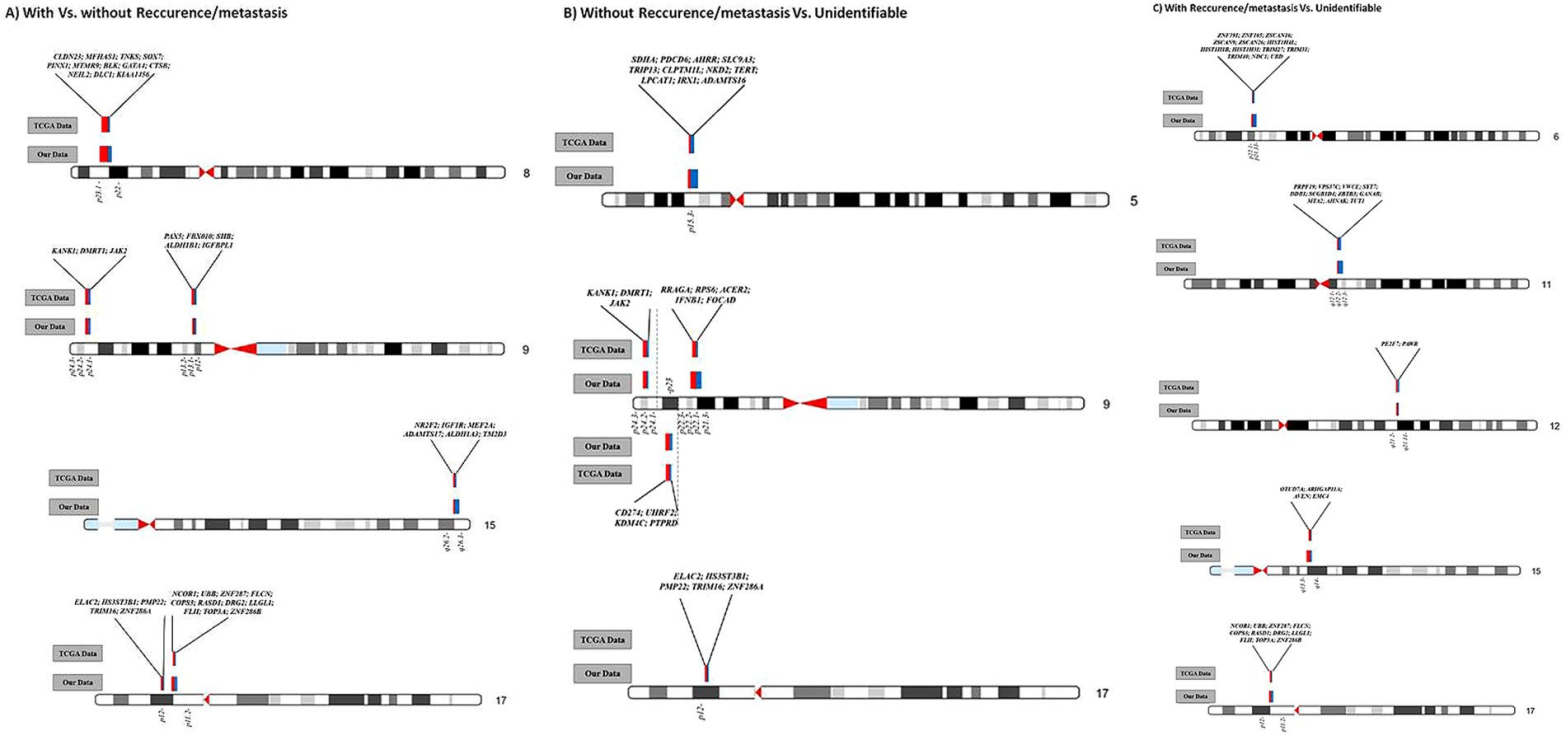
Ideogram with chromosomal regions used by predictive genomic model and the highlighted candidate genes in these regions. (**A**) In patients with vs. without recurrence/metastasis - first phase of the predictive model; (**B**) in patients without recurrence and those unidentifiable - second phase of the predictive model; (**C**) in patients with recurrence and those unidentifiable - third phase of the predictive model. Blue represents the proportion of copy number gains and red represents the copy number losses identified in these specific chromosomal regions both in our HNSCC patients and in TCGA database.

In the specific chromosomal regions of chromosomes 5p, 6p, 8p, 9p, 11q, 12q, 15q and 17p that were used in this three-phased predictive model are mapped important genes for the carcinogenesis process. Through the analysis of the genes mapped in these chromosomal regions using UCSC genome browser (https://genome.ucsc.edu/) and GeneCards - Human gene database (http://www.genecards.org/) we identified some potential candidate genes connected with signaling pathways that control processes associated with tumorigenesis (Fig. 3).

### Validation of the predictive model for recurrence and metastasis development using a TCGA cohort

The usefulness of the developed predictive model based on CNAs was validated using TCGA data from 95 HNSCC, which presented full information regarding their vital status.

Our three-phase model, when applied to TCGA cohort presented an accuracy of 59.4% CI95% [38.9; 77.8] %, correct prediction of patients without recurrence of 74.7% CI95% [33.3; 100]%, correct prediction of patients with recurrence of 55.1% CI95% [16.7; 100]% and correct prediction of patients unidentified of 48.5% CI95% [0; 100]%.

## Discussion

The development and progression of HNSCC is significantly correlated with the accumulation of genomic alterations, allowing cells to escape homeostatic controls that suppress inappropriate proliferation, which result not only in increased proliferation but also in, metabolic changes, genetic instability, induction of angiogenesis, resistance to cell death and increased migratory capacity^9^. Identification of genome-wide high resolution DNA copy number changes through array-CGH has been applied to a wide range of tumors including HNSCC^10–12^. Genomic instability is a common characteristic of cancer cells, with aneuploidy and large-scale DNA rearrangements being frequently observed^13–15^. However, the relevant chromosomal variations and genes that play a central role in HNSCC development and progression as well as in recurrence and metastasis development are not still fully elucidated. In this study we observed several copy number gains and losses in all chromosomes, with chromosomes 3, 5, 7, 8, 9, 11, 12, 14, 15, 16, 17, 18, 19, 20 and 22 being the most frequently altered in our HNSCC cohort (Fig. 1). These results revealed the great genomic complexity that underlies HNSCC. Additionally, these tumors exhibit great heterogeneity in their clinical behavior that cannot currently be predicted using only the available set of clinical markers; therefore, the development of a prognosis predictive model is a novel and promising strategy to increase the HNSCC survival rate and improve the quality of life of the patients. We genomically characterized HNSCC through array-CGH technology, highlighting specific chromosomal alterations. The identified genomic signature was used to build a predictive statistical model of recurrence and metastasis development (Fig. 2). This predictive multivariate model presented average accuracy higher than 80% and was validated in a TCGA cohort. This model comprises several upstream and downstream members of signaling pathways that lead to an increase in the cell proliferation and invasion (Fig. 3). Components of PI3K/Akt, mTOR, Wnt, Hedgehog, Hippo, Notch, MAP/ERK, were identified as affected in our cohort since several downstream nuclear targets of these signaling pathways are deregulated in the chromosomal regions used by the developed predictive model, such as, *NKD2* (5p15.33), *SOX7* (8p23.1), *RRAGA* (9p22.1) *KANK1* (9p24.3), *JAK2* (9p24.1), *LLGL1* (17p12.2) and *FLCN* (17p11.2). Likewise, genes related to regulation of telomerase, cytoskeletal, metabolism and DNA repair were also frequently altered in tumors and used in this predictive model, namely *SDHA* and *LPCAT1* (5p15.33), MDC1 (6p21.33), *PINX1* (8p23), *ACER2* (9p22.1), PRPF19 (6p21.33), *AVEN* (15q14) and *FLII* (17p11.2). As consequence of tumor progression, neoplastic cells become more migratory and develop the capability to invade surrounding tissues, which is accompanied by alterations in adhesion, cell polarity, cytoskeletal dynamics and morphology^9^. Cell growth is coordinated with metabolic processes involved in the synthesis of macromolecules, thus, cancer cells display metabolic plasticity, altering their metabolic profile during tumorigenesis and metastasis. Altogether, these results suggest a specific set of chromosomal regions and genes that seem to have an important role in the development and prediction of HNSCC recurrence/metastasis. This model could also help in the design of targeted therapies; however, cancer cells seem to develop resistance to inhibition of a particular signaling pathway by expressing alternate protein isoforms or up-regulating compensatory pathways; thus, cancer therapeutic strategies should involve targeting simultaneously multiple deregulated signaling pathways. The observed genomic instability of HNSCC reflects a failure of checkpoint signaling and/or DNA repair mechanisms, denoting the clear need for further research in this area to establish a precise link between these highlighted specific candidate genes and the HNSCC recurrence/metastasis and, consequently with patients’ prognosis.

It is important to stress some limitations of this study, namely the fact that our cohort presented a relatively reduced clinical follow-up time (range from 6 to 64 months), so, some patients with a genomic profile similar to those with recurrence/metastasis could be incorrectly classified in the first phase of the predictive model only because the patients were not followed up enough time to be diagnosed with recurrence/metastasis. This scenario would justify the great majority of unidentified patients. Another limitation is the fact that patients with different anatomic tumors in the head and neck region were analyzed as a homogenous entity, but these tumors were indeed already described as clinical and molecular different entities. In the future, this predictive model should be tested in larger cohorts of different populations of the different head and neck anatomic subsites. Further studies and larger follow-up times should be performed to better characterize the unidentified patients. The validation of this predictive model in the TCGA cohort presented overall reduced quality comparatively to our cohort, which could be due to the fact that we are testing a model specifically developed to array-CGH data in results obtained with a different platform, SNP- microarray. Although, this result may indicate a lack of generalizability, the results obtained in our cohort in the train set are quite similar to the test set, suggesting a good capacity for the model to generalized results. Notice, that the comparison of results in the same cohort permit to control for the fact that the features in the TGCA cohort were not exactly measured as in our cohort. In addition, the two cohorts are not well matched presenting different strata which could not be individually treated because of the small sample size. As so, these different factors impact on the results obtained in the TGCA cohort and may explain the high reduction of accuracy observed. Larger cohorts would also permit the use of more genes, as well as the fine tuning of the set of genes that are more important to the prediction and thus more involved in the development of metastasis/recurrence. The clinical application of this genomic predictive model is promising since it is possible to identify newly diagnosed HNSCC patients with risk of development of recurrence/metastasis and, in this sense, monitor them closely, avoiding or performing early detection of the recurrences and even provide more aggressive and personalized treatment in order to reduce the morbidity and mortality associated with this disease. The complexity of the cancer signaling pathways presents a significant challenge to the development of targeted therapies due to the redundancy of the pathways that control cell proliferation and survival, the crosstalk between pathways, and the feedback inhibition mechanisms that cause pathway reactivation; however, we highlighted in this study several chromosomal regions and genes that could be good candidates for targeted therapy studies.

Since HNSCC has a poor overall prognosis with a high tendency to recur at the primary site and to involve the cervical lymph nodes, this predictive model for recurrence and metastasis development may pave the way to a more practical and individualized patient management and targeted drug design.

## Electronic supplementary material

Supplementary Tables 1, 2 and 3

## References

[1] Ferlay J (2010). Estimates of worldwide burden of cancer in 2008: GLOBOCAN 2008. International journal of cancer.

[2] Thariat J (2015). Integrating genomics in head and neck cancer treatment: Promises and pitfalls. Critical reviews in oncology/hematology.

[3] Worsham MJ (2011). Identifying the risk factors for late-stage head and neck cancer. Expert review of anticancer therapy.

[4] Argiris A, Karamouzis MV, Raben D, Ferris RL (2008). Head and neck cancer. Lancet.

[5] Vermorken JB (2008). Platinum-based chemotherapy plus cetuximab in head and neck cancer. The New England journal of medicine.

[6] Jerjes W (2010). Clinicopathological parameters, recurrence, locoregional and distant metastasis in 115 T1-T2 oral squamous cell carcinoma patients. Head & neck oncology.

[7] Worsham MJ, Ali H, Dragovic J, Schweitzer VP (2012). Molecular characterization of head and neck cancer: how close to personalized targeted therapy?. Molecular diagnosis & therapy.

[8] Pinto-Leite R (2014). Genomic characterization of three urinary bladder cancer cell lines: understanding genomic types of urinary bladder cancer. Tumour Biol.

[9] Sever, R. & Brugge, J. S. Signal transduction in cancer. *Cold Spring Harbor perspectives in medicine* **5**, 10.1101/cshperspect.a006098 (2015). 10.1101/cshperspect.a006098 PMC4382731 25833940

[10] Cha J-D, Kim HJ, Cha I-H (2011). Genetic alterations in oral squamous cell carcinoma progression detected by combining array-based comparative genomic hybridization and multiplex ligation-dependent probe amplification. Oral Surgery, Oral Medicine, Oral Pathology, Oral Radiology, and Endodontology.

[11] Chen YJ (2004). Genome-wide profiling of oral squamous cell carcinoma. The Journal of pathology.

[12] Sparano A (2006). Genome-wide profiling of oral squamous cell carcinoma by array-based comparative genomic hybridization. The Laryngoscope.

[13] Ribeiro, I. P. *et al*. Genetic gains and losses in oral squamous cell carcinoma: impact on clinical management. *Cell Oncol (Dordr)*, 10.1007/s13402-013-0161-5 (2014). 10.1007/s13402-013-0161-5 PMC13004429 24353162

[14] Ribeiro, I. P. *et al*. Genetic imbalances detected by multiplex ligation-dependent probe amplification in a cohort of patients with oral squamous cell carcinoma-the first step towards clinical personalized medicine. *Tumour Biol*, 10.1007/s13277-014-1614-9 (2014). 10.1007/s13277-014-1614-9 24477574

[15] Ribeiro, I. P. *et al*. WT1, MSH6, GATA5 and PAX5 as epigenetic oral squamous cell carcinoma biomarkers - a short report *Cellular Oncology*, 10.1007/s13402-016-0293-5 (2016). 10.1007/s13402-016-0293-5 PMC13001870 27491556

