# Supplementary material for: Genomic predictive model for recurrence and metastasis development in head and neck squamous cell carcinoma patients
Source: Sci Rep. 2017 Oct 24;7:13897. doi: 10.1038/s41598-017-14377-x (PMC5654944; doi:10.1038/s41598-017-14377-x)
Supplement: Supplementary file 1 — Supplementary Tables 1, 2 and 3 [file 41598_2017_14377_MOESM1_ESM.pdf]

Title: Genomic predictive model for recurrence and metastasis development in head and neck squamous cell carcinoma patients

Ilda Patrícia Ribeiro[1,2], Francisco Caramelo[3], Luísa Esteves[1], Joana Menoita[1], Francisco Marques[2,4,5], Leonor Barroso[6], Jorge Miguéis[7], Joana Barbosa Melo[1,2], Isabel Marques Carreira[1,2†]

Supplementary Table 1. List of candidate genes identified in the chromosomal regions used by the first phase of predictive model: identification of patients with vs. without recurrence/metastasis.

	Candidate genes	Biological function	Related pathways (GeneCards)
**8p23.1-p22**	*CLDN23*	Signal transduction	Blood-Brain Barrier and Immune Cell Transmigration: VCAM-1/CD106 Signaling Pathways and Tight junction
	MFHAS1	Potential oncogene	-
	TNKS	Wnt signaling pathway, telomere length and vesicle trafficking	Nicotinate and nicotinamide metabolism and HIV Life Cycle
	SOX7	Tumorigenesis	Wnt Signaling and ERK Signaling
	PINX1	Tumor suppressor	Regulation of Telomerase
	MTMR9	Cell proliferation	-
	BLK	Cell proliferation and differentiation	CXCR4-mediated signaling events and B cell receptor signaling pathwa
	GATA4	Transcriptional regulation	DREAM Repression and Dynorphin Expression and Cardiac conduction
	CTSB	Associated with esophageal adenocarcinoma	Immune System and Toll-Like receptor Signaling Pathways
	NEIL2	Base excision repair of DNA	Recognition and association of DNA glycosylase with site containing an affected pyrimidine and Telomere C-strand (Lagging Strand) Synthesis
	DLC1	Tumor suppressor	G-protein signaling_Regulation of CDC42 activity and Regulation of RhoA activity
	KIAA1456	Tumor suppressor	tRNA processing and Gene Expression
**9p13.2-p12**	*PAX5*	Differentiation	Transcriptional misregulation in cancer
	FBX010		-
	SHB	Angiogenesis; Apoptosis	EPH-Ephrin signaling and Development VEGF signaling via VEGFR2 - generic cascades
	ALDH1B1	Alcohol metabolism	Metabolism and Cytochrome P450 - arranged by substrate type
	IGFBPL1	Putative tumor suppressor	-
**9p24.3p24.1**	*KANK1*	Putative tumor suppressor	PI3K/Akt signaling
	DMRT1	Putative tumor suppressor	-
	JAK2	Cell growth, differrentiation and histone modifications	Interferon gamma signaling and RET signaling
**15q26.2-q26.3**	*NR2F2*	Transcriptional regulation	Oct4 in Mammalian ESC Pluripotency and Regulation of lipid metabolism by Peroxisome proliferator-activated receptor alpha (PPARalpha)
	IGF1R	Anti-apoptotic agent by enhancing cell survival	IL-2 Pathway and Development Ligand-independent activation of ESR1 and ESR2
	MEF2A	Cell growth control and apoptosis	Activated TLR4 signalling and Immune System
	ADAMTS17	Peptidase activity and metalloendopeptidase activity	O-glycosylation of TSR domain-containing proteins and HIV Life Cycle
	ALDH1A3	Aldehyde dehydrogenase (NAD) activity	Drug metabolism - cytochrome P450 and Tyrosine metabolism.
	TM2D3	Regulation of cell death or proliferation signal cascades	-
**17p12**	*ELAC2*	Mediated growth arrest	tRNA processing and rRNA processing in the mitochondrion
	HS3ST3B1	Epithelial-mesenchymal transition	Cytochrome P450 - arranged by substrate type and Metabolism
	PMP22	Growth regulation	a6b1 and a6b4 Integrin signaling and Neural Crest Differentiation
	TRIM16	Cell growth, differentiation and pathogenesis	-
	ZNF286A	Transcriptional regulation	Gene Expression
**17p12-p11.2**	*NCOR1*	Chromatin remodeling and repression of transcription	Regulation of nuclear SMAD2/3 signaling and BMAL1-CLOCK,NPAS2 activates circadian gene expression
	UBB	Involved in the maintenance of chromatin structure, the regulation of gene expression, and the stress response.	Interferon gamma signaling and Activated TLR4 signalling
	ZNF287	Transcriptional regulation	Gene Expression
	FLCN	Putative tumor suppressor	ErbB signaling pathway and mTOR signaling pathway
	COPS3	Signal transduction	Vesicle-mediated transport and Transcription-Coupled Nucleotide Excision Repair (TC-NER)
	RASD1	Cell morphology, growth and cell-extracellular matrix interactions	Neuroscience and MAP Kinase Signaling.
	DRG2	Cell growth and differentiation	-
	LLGL1	Cytoekeletal network	Tight junction and Hippo signaling pathway
	FLII	Regulation of cytoekeletal rearrangements	Cytoskeletal Signaling
	TOP3A	Controls and alters the topologic states of DNA during transcription	Meiosis and Cell Cycle Checkpoints
	ZNF286B	Transcriptional regulation	-

Supplementary Table 2. List of candidate genes identified in the chromosomal regions used by the second phase of predictive model: identification of patients without recurrence/metastasis. vs. unidentifiable

	Candidate genes	Biological function	Related pathways (GeneCards)
**5p15.33-p15.32**	*SDHA*	Putative tumor suppressor	Metabolism and Citrate cycle (TCA cycle)
	PDCD6	May inhibit KDR/VEGFR2-dependent angiogenesis; the function involves inhibition of VEGF-induced phosphoprylation of the Akt signaling pa	-
	AHRR	Cell growth and differentiation	Regulation of lipid metabolism by Peroxisome proliferator-activated receptor alpha (PPARalpha) and Metabolism
	SLC9A3	Signal transduction	Transport of glucose and other sugars, bile salts and organic acids, metal ions and amine compounds and Protein digestion and absorption.
	TRIP13	Putative role in early-stage non-small cell lung cancer	-
	CLPTM1L	Enhances cisplatin-mediated apoptosis	-
	NKD2	Negative regulators of Wnt receptor signaling	Wnt Signaling Pathway and Pluripotency and Wnt / Hedgehog / Notch
	TERT	Cellular senescence and oncogenesis	HTLV-I infection and Chromosome Maintenance
	LPCAT1	Progression of oral squamous cell, prostate, breast, and other human cancers	Metabolism and Acyl chain remodelling of PE
	IRX1	Tumor suppressor in gastric and head and neck cancers	-
	ADAMTS16	Metalloendopeptidase activity	O-glycosylation of TSR domain-containing proteins and HIV Life Cycle
**9p22.3-p21.3**	*RRAGA*	Inhibitor of TNF-alpha functions, affecting cell death	mTOR signalling and TP53 Regulates Metabolic Genes
	RPS6	Cell growth and proliferation	Activation of the mRNA upon binding of the cap-binding complex and eIFs, and subsequent binding to 43S and Metabolism
	ACER2	Cell proliferation and survival	Metabolism and sphingosine and sphingosine-1-phosphate metabolism
	IFNB1	Cell differentiation and anti-tumor defenses	Interferon gamma signaling and Regulation of nuclear SMAD2/3 signaling
	FOCAD	Potential tumor suppressor in gliomas	-
**9p24.3p24.1**	*KANK1*	Putative tumor suppressor	PI3K/Akt signaling
	DMRT1	Putative tumor suppressor	-
	JAK2	Cell growth, differrentiation and histone modifications	Interferon gamma signaling and RET signaling
**9p24.1-p23**	*CD274*	Prognostic value in colon cancer and renal cell carcinoma	Immune System and IgA-Producing B Cells in the Intestine
	UHRF2	Cell cycle regulation	
	KDM4C	Regulation of gene expression and chromosome segregation	Activated PKN1 stimulates transcription of AR (androgen receptor) regulated genes KLK2 and KLK3 and Chromatin organization
	PTPRD	Cell growth, differentiation, mitotic cycle, and oncogenic transformation	Transmission across Chemical Synapses and Protein-protein interactions at synapses.
**17p12**	*ELAC2*	Mediated growth arrest	tRNA processing and rRNA processing in the mitochondrion
	HS3ST3B1	Epithelial-mesenchymal transition	Cytochrome P450 - arranged by substrate type and Metabolism
	PMP22	Growth regulation	a6b1 and a6b4 Integrin signaling and Neural Crest Differentiation
	TRIM16	Cell growth, differentiation and pathogenesis	-
	ZNF286A	Transcriptional regulation	Gene Expression

Supplementary Table 3. List of candidate genes identified in the chromosomal regions used by the third phase of predictive model: identification of patients with recurrence /metastasis. vs. unidentifiable

	Candidate genes	Biological function	Related pathways (GeneCards)
**6p22.1-p21.33**	*ZNF391; ZNF165; ZSCAN16; ZSCAN9; ZSCAN26*	Transcriptional regulation	-
	HIST1H4L	Transcription regulation, DNA repair, DNA replication and chromosomal stability	Mitotic Prophase and Activated PKN1 stimulates transcription of AR (androgen receptor) regulated genes KLK2 and KLK3
	HIST1H1B	Regulator of individual gene transcription through chromatin remodeling, nucleosome spacing and DNA methylation	Apoptosis induced DNA fragmentation and Regulation of activated PAK-2p34 by proteasome mediated degradation
	HIST1H3I	Transcription regulation, DNA repair, DNA replication and chromosomal stability	Mitotic Prophase and Immune System
	TRIM27	Transcriptional repressor activity; Apoptosis	-
	TRIM31	Negative regulator of cell growth	Interferon gamma signaling and Immune System
	TRIM40	Negative regulator against inflammation and carcinogenesis	-
	MDC1	Cell cycle checkpoints in response to DNA damage	DNA Double Strand Break Response and ATM Pathway
	UBD	Apoptosis	PEDF Induced Signaling and Beta-Adrenergic Signaling
**11q12.1-q12.3**	*PRPF19*	Cell survival and DNA repair	mRNA Splicing - Major Pathway and Transcription-Coupled Nucleotide Excision Repair (TC-NER)
	VPS37C	Cell growth and differentiation	Vesicle-mediated transport and HIV Life Cycle
	VWCE	Target for chemoprevention of hepatocellular carcinoma	-
	SYT7	Associated with prostate cancer.	Protein-protein interactions at synapses and Transmission across Chemical Synapses
	DDB1	Damaged DNA binding	Toll-like receptor signaling pathway and Nucleotide excision repair
	SCGB1D4	Involved in the regulation of chemotactic cell migration and invasion	-
	ZBTB3	Transcriptional regulation	-
	GANAB	Associated to lung tumor	Transport to the Golgi and subsequent modification and Calnexin/calreticulin cycle
	MTA2	Transcriptional regulation (It is closely related to another member of this family, a protein that has been correlated with the metastatic potential of certain carcinomas)	Activated PKN1 stimulates transcription of AR (androgen receptor) regulated genes KLK2 and KLK3 and RNA Polymerase I Promoter Escape
	AHNAK	Tumor metastasis	-
	TUT1	Regulation of gene expression and cell proliferation	-
12q21.2-q21.31	*E2F7*	Regulation of cell cycle progression	TP53 Regulates Transcription of Cell Cycle Genes and Gene Expression
	PAWR	Tumor suppressor	-
15q13.3-q14	*OTUD7A*	Putative tumor suppressor	Ovarian tumor domain proteases and Metabolism of proteins
	ARHGAP11A	Cell-cycle arrest and apoptosis	Signaling by GPCR and p75 NTR receptor-mediated signalling
	AVEN	Apoptosis	Apoptosis and Autophagy
	EMC4	Anti-apoptotic activity	-
**17p12-p11.2**	*NCOR1*	Chromatin remodeling and repression of transcription	Regulation of nuclear SMAD2/3 signaling and BMAL1-CLOCK,NPAS2 activates circadian gene expression
	UBB	Involved in the maintenance of chromatin structure, the regulation of gene expression, and the stress response.	Interferon gamma signaling and Activated TLR4 signalling
	ZNF287	Transcriptional regulation	Gene Expression
	FLCN	Putative tumor suppressor	ErbB signaling pathway and mTOR signaling pathway
	COPS3	Signal transduction	Vesicle-mediated transport and Transcription-Coupled Nucleotide Excision Repair (TC-NER)
	RASD1	Cell morphology, growth and cell-extracellular matrix interactions	Neuroscience and MAP Kinase Signaling.
	DRG2	Cell growth and differentiation	-
	LLGL1	Cytoekeletal network	Tight junction and Hippo signaling pathway
	FLII	Regulation of cytoekeletal rearrangements	Cytoskeletal Signaling
	TOP3A	Controls and alters the topologic states of DNA during transcription	Meiosis and Cell Cycle Checkpoints
	ZNF286B	Transcriptional regulation	-
